# Is General Anesthesia Safe for a Child with Acute Upper Respiratory Tract Infection? A Narrative Review

**DOI:** 10.3390/pediatric17050106

**Published:** 2025-10-13

**Authors:** Jowita Rosada-Kurasińska, Alicja Bartkowska-Śniatkowska, Anna Wiernik, Bartłomiej Kociński, Małgorzata Grześkowiak

**Affiliations:** 1 Department of Paediatric Anaesthesiology and Intensive Therapy, Poznan University of Medical Sciences, 61-701 Poznań, Poland; asniatko@ump.edu.pl (A.B.-Ś.);; 2 Department of Paediatric Cardiac Surgery, Poznan University of Medical Sciences, 61-701 Poznań, Poland; 3 Department of Anaesthesiology and Intensive Therapy Teaching, Poznan University of Medical Sciences, 61-701 Poznań, Poland; mgrzesko@ump.edu.pl

**Keywords:** general anesthesia, children, acute respiratory infection

## Abstract

Anesthesia for children with a current respiratory infection or a history of infection within the last two weeks is always associated with a high risk of respiratory complications. However, this risk decreases significantly when the interval between the last symptoms of the infection and the planned anesthesia and medical procedure is extended to at least 2–4 weeks. The most common adverse events include bronchospasm, laryngospasm, and apnea. For children requiring unplanned procedures—such as emergency, urgent, or immediate general anesthesia—guidelines should be followed to maximize their safety and minimize the risk of complications.

## 1. Introduction

Upper respiratory tract infection (URTI) is defined as a self-limiting inflammation and edema of the upper airway mucosa caused by viral or bacterial pathogens [1]. In most literature reports, URTI is diagnosed when at least two of the following symptoms are present: runny nose, sore throat, sneezing, nasal congestion, malaise, cough, or fever exceeding 38 °C. URTI is a common condition in children, particularly in early childhood. Children under 4 years of age experience an average of up to eight respiratory tract infections per year, with the incidence decreasing with age. Moreover, URTI shows a distinct seasonal pattern, with a higher incidence during colder months [2,3]. Pediatric URTIs represent a significant social and economic burden due to increased healthcare utilization, school absenteeism, and parental loss of working days

Although most URTI symptoms are mild and self-limiting, they can have a significant impact on perioperative preparation. Concomitant upper respiratory tract infection and exposure to chronic respiratory irritants are predisposing factors for adverse respiratory events during anesthesia in children. Such events are a major contributor to increased morbidity and mortality associated with pediatric anesthesia [4].

The common cold is most often caused by rhinoviruses, accounting for up to 80% of cases. Other pathogens include respiratory syncytial virus (RSV), influenza virus, parainfluenza virus, coronavirus, adenovirus, and metapneumovirus [2]. Many experts regard RSV infection as the most serious respiratory illness requiring special attention, thorough evaluation, and careful perioperative planning, particularly in infants and children under 2 years of age [5]. RSV is typically the leading cause of severe respiratory illness in children, especially among immunocompromised patients, those with comorbidities, and young infants. It is a major contributor to pediatric hospitalizations and remains one of the leading causes of infant mortality worldwide. RSV can increase airway hyperresponsiveness and is recognized as a causative factor in the development of asthma [6].

A current concern is infection caused by COVID-19. Severe acute respiratory syndrome coronavirus 2 (SARS-CoV-2) infection presents with a wide range of symptoms, from upper respiratory tract infection (URTI) manifestations, fever, and gastrointestinal symptoms to severe acute respiratory distress syndrome (ARDS), shock, multiorgan failure, and even death. The incidence increases with age. Many patients, particularly younger children and those who are vaccinated, remain asymptomatic; however, children under 4 years of age are at the highest risk of hospitalization, intensive care unit (ICU) admission, and mortality [7]. Peterson et al. demonstrated that children with COVID-19 have a higher incidence of hypoxemia and perioperative complications during airway management under general anesthesia [8]. These patients were 2.7 times more likely to experience hypoxemia during tracheal intubation or extubation. Moreover, the severity of hypoxemia was greater, and complications such as laryngospasm occurred more frequently in children with COVID-19 [8]. In the absence of national or local guidelines, the literature recommends postponing surgery for 2 weeks after resolution of symptoms in uncomplicated URTI, and for 4 weeks following severe URTI symptoms (such as fever above 40 °C). A persistent cough, which may continue for several weeks, should be considered an indicator of ongoing bronchial hyperreactivity and not overlooked [1]. Although these recommendations primarily apply to COVID-19 infections, they can be extended to respiratory infections of other etiologies as well.

URTI triggers an inflammatory response characterized by infiltration of immune cells into the respiratory mucosa and bronchial smooth muscle. This process is associated with bronchial hyperreactivity, which can lead to bronchospasm and laryngospasm [1]. The mechanism of bronchospasm involves the release of acetylcholine from postganglionic parasympathetic nerve endings. Acetylcholine binds to M3 muscarinic receptors, which belong to the group of metabotropic receptors acting through G proteins, ultimately causing contraction of bronchial smooth muscle and narrowing of the airways.

Stimulation of these receptors directly affects bronchial smooth muscle, leading to contraction. At the same time, acetylcholine also binds to another type of muscarinic receptor, M2, which inhibits further acetylcholine release and thereby reduces the effect on the initially stimulated M3 receptors. Following administration of an M2 receptor antagonist, such as gallamine, M3 receptor activity may predominate by up to tenfold, resulting in pronounced bronchoconstriction. In contrast, administration of an M2 receptor agonist, such as pilocarpine, enhances M2 receptor activity and can reduce bronchoconstriction by up to 85%. M2 receptors activate Gi proteins, which inhibit adenylyl cyclase activity. The resulting decrease in cAMP levels enhances potassium channel conductance, leading to hyperpolarization of the cell membrane and inhibition of voltage-gated calcium channels, thereby reducing calcium influx and modulating smooth muscle tone.

As early as 1979, Empey observed that bronchial hyperresponsiveness following viral infections in children may persist for up to 4 to 6 weeks [9].This effect is likely related to dysfunction of M2 muscarinic receptors. However, this mechanism may not be exclusively infection-related; it can also occur in the context of allergic conditions, possibly through eosinophil activation and inhibition of interferon gamma production [9,10].

What exactly occurs during viral infections? Neuraminidase is an enzyme that cleaves sialic acid residues from glycoproteins, thereby facilitating viral entry into host cells and promoting spread to adjacent cells [11]. Sialic acid, however, is also a structural component of the M2 muscarinic receptor. When a virus bearing neuraminidase on its surface contacts the receptor, sialic acid is removed, altering the receptor’s structure and leading to its dysfunction. This reaction occurs exclusively with the type 2 muscarinic receptor and not with other muscarinic receptor subtypes. Clinically, this dysfunction reduces the bronchi’s ability to relax, and the impaired state may persist for up to 6 weeks [12].

From the perspective of gas exchange, the most important structure in respiration is the alveolar–capillary barrier. The alveolar surface is lined by epithelial cells, while the inner surface of the adjacent blood vessel is lined by endothelial cells; both layers rest on a shared basement membrane.

Both sides of the alveolar–capillary barrier can contribute to the development of acute lung injury. On the airway side, injury may result from mechanical stress, including barotrauma, volutrauma, and atelectasis caused by mechanical ventilation, as well as from microbiological factors that directly damage the epithelium. On the vascular side, the endothelium is covered by the glycocalyx, which maintains vascular integrity and limits permeability. Among the perioperative factors that damage the glycocalyx, the most significant are hypervolemia, blood product transfusion, ischemia–reperfusion injury, sepsis, and, in particular, tumor necrosis factor (TNF-α) and lipopolysaccharides (LPS) [13]. To prevent these complications, appropriate management should include both a lung-protective ventilation strategy and optimized fluid therapy. Historically, tidal volumes (TV) exceeding 12 mL/kg were recommended—approximately twice the physiological TV of 6 mL/kg in humans. Current guidelines for ventilation in older children recommend a TV of 6 mL/kg along with the consistent use of positive end-expiratory pressure (PEEP) [14]. Likewise, to prevent glycocalyx shedding and the resulting increase in endothelial permeability, which allows fluid and solutes to enter the alveoli, fluid therapy should be rational and tailored to the actual fluid requirements. Early implementation of preventive strategies in patients at risk of developing acute respiratory distress syndrome (ARDS), even during the preoperative and intraoperative periods, through appropriate ventilation management, can significantly reduce its incidence and severity [15].

It is important to note that routine microbiological diagnostics in children during the preoperative period are not required, as the results rarely influence anesthetic management. However, most children, particularly those with severe illness, undergo such testing earlier during hospitalization in pediatric departments, which substantially facilitates the implementation of appropriate preventive and therapeutic strategies.

## 2. Preparing a Child with a Respiratory Infection for Anesthesia

Children with respiratory infections are at particularly high risk of perioperative respiratory adverse events (PRAEs) [1]. Therefore, a thorough preoperative assessment is essential, taking into account both the type of respiratory infection and the child’s individual risk factors. The common cold is characterized by nasal discharge, sneezing, sore throat, and cough. Reported symptom frequencies include tearing (66%), nasal congestion (37%), sneezing (29%), productive cough (26%), sore throat (8%), and fever (8%) [2]. Bronchitis and tracheitis may present with a dry cough and wheezing. Importantly, children with current or recent URTI—within two weeks before anesthesia and surgery—are at increased risk of perioperative respiratory events such as laryngospasm, bronchospasm, desaturation, and respiratory arrest [4,7,8]. These complications result from an inflammatory response marked by immune cell infiltration into the respiratory mucosa and bronchial smooth muscle. This process is associated with bronchial hyperreactivity, increased mucus production, nasal congestion, and tracheal edema, all of which elevate the risk during airway management. These changes may persist for up to four weeks after infection. Additional factors that further increase the risk in this patient group include age under two years, history of prematurity, exposure to passive smoking, pre-existing respiratory diseases, planned airway surgery, and the use of an endotracheal tube or other airway prosthesis [16,17].

As a simple decision-making tool, the COLDS score can be used for the preanesthetic evaluation of children with upper respiratory tract infections. This nonstandardized scale is designed to convey overall risk rather than to provide statistically validated probabilities of specific complications. Each of the five COLDS categories is assigned one, two, or five points, for a total possible score ranging from 5 to 25; higher scores indicate greater perianesthetic risk [18] (Table 1). The COLDS scoring system underscores that no anesthetic protocol is entirely risk-free for children with upper respiratory infections. It also improves specificity by minimizing situations in which different combinations of category scores result in the same total score. The COLDS score is intended primarily for use in elective procedures when the diagnosis of upper respiratory infection (URI) is clear and unambiguous. As a heuristic decision aid rather than a statistically validated risk model, a particular COLDS score may be acceptable in some clinical settings but not in others. The authors emphasize the usefulness of identifying “red flags” to help align institutional or clinician risk tolerance with COLDS results. Any category assigned the maximum of 5 points should be regarded as a red flag, cautioning against proceeding with anesthesia. Thresholds can be adjusted to limit the number of red flags: a total score of 12 or less corresponds to at most one red flag; a score of 16 or less corresponds to no more than two; and a score up to 19 allows for a maximum of three red flags. This framework enables flexible, individualized decision-making using the COLDS score rather than applying a rigid universal cutoff.

Anesthesia for ear, nose, and throat (ENT) procedures represents a distinct consideration. These surgeries, such as myringotomy or tonsillectomy, are often performed to eliminate the underlying source of infection. They may proceed even in the presence of mild upper respiratory tract infection symptoms, as in such cases the potential benefits frequently outweigh the increased risk of perioperative respiratory adverse events [19].

## 3. Preoperative Management

### 3.1. Premedication

No studies have specifically evaluated the use of sedative premedication to reduce adverse respiratory events in children with URTI [1]. In children presenting with URTI symptoms, benzodiazepines should not be used as first-line premedication due to the increased risk of respiratory complications [3]. For patients requiring premedication, alpha-2 adrenergic receptor agonists, such as clonidine or dexmedetomidine, appear to be a safer alternative, as they do not exert respiratory depressant effects. A large randomized clinical trial demonstrated that intranasal midazolam used for premedication was associated with a higher incidence of perioperative respiratory adverse events, whereas intranasal dexmedetomidine was associated with a reduced incidence of such events [20]. An effective and increasingly popular approach for alleviating preoperative anxiety and fear in pediatric patients involves non-pharmacological distraction techniques, such as interactive games on smartphones or tablets, the use of toys for younger children, or oral administration of 12% or 25% sucrose solution in neonates and infants [21]. A separate consideration is the preoperative use of bronchodilators. β_2_-adrenergic agonists such as salbutamol may help reduce perioperative respiratory adverse events, including bronchospasm and severe coughing, in children with URTI or asthma [22]. In a study by von Ungern-Sternberg, administration of nebulized albuterol 10–30 min before surgery significantly reduced the incidence of perioperative bronchospasm and severe coughing in children with a “moist cough” compared to controls. Consequently, the use of inhaled salbutamol is recommended in children with current or recent (<2 weeks) URTI 10–30 min before induction, at a dose of 2.5 mg for children weighing up to 20 kg and 5 mg for those weighing more than 20 kg [23].

### 3.2. General Aesthesia

Currently, there are no evidence-based guidelines or recommendations to assist pediatric anesthesiologists in selecting the type of anesthetic induction technique that could reduce or prevent perioperative respiratory adverse events (PRAEs) [24].

Intravenous induction with propofol is associated with a significant reduction in adverse airway reflexes compared with inhalational induction [16]. Owing to its ability to suppress airway reflexes, propofol appears to be an ideal agent for anesthesia induction in children at increased risk of respiratory adverse events. However, its bronchodilatory effect is relatively weak [25].

Inhaled anesthetics exert beneficial effects on the airways due to their bronchodilatory properties, although they have limited efficacy in suppressing airway reflexes [26]. These agents are recommended for the management of severe intraoperative bronchospasm or acute asthma but are not suitable for treating acute laryngospasm. Among volatile anesthetics, sevoflurane is preferred because of its potent bronchodilatory effect [3,26]. In contrast, desflurane should be avoided, as it increases airway resistance and has an irritating odor, both of which are associated with a significantly higher risk of adverse airway reflexes, particularly in children under 12 years of age and those presenting with upper respiratory tract infection symptoms.

Although some studies suggest that total intravenous anesthesia with propofol is associated with a reduced risk of perioperative respiratory adverse events compared with inhalational anesthesia using sevoflurane [3,24], certain patients may still benefit from inhalational induction—for example, those with needle phobia or a history of difficult intravenous access [24].

The concentration of oxygen in the respiratory gas mixture during anesthesia induction in children is another important consideration. The instinctive tendency is to use the highest possible oxygen concentration to permit unrestricted airway maneuvers without the risk of desaturation. However, it is well established that high oxygen concentrations, particularly in patients at the extremes of age, can cause irreversible and serious consequences. These include not only macroscopic changes such as bronchial dilation and retinopathy, but also paradoxical effects on the respiratory system, such as severe atelectasis and a consequent reduction in functional residual capacity (FRC). In addition, high oxygen levels can induce apoptosis in oligodendrocytes and result in central nervous system (CNS) injury. For these reasons, the recommended oxygen concentration during induction should not exceed 80%, be appropriately reduced during maintenance, and increased again during emergence—without ever reaching 100% [27].

The direct effect of neuromuscular blocking agents on laryngeal or bronchial spasm in children, particularly in the setting of acute infection, has not yet been described in the literature. However, the functional mechanisms of bronchial activity have been well characterized in animal models, where two key processes—contraction and relaxation—are regulated by numerous receptors, including muscarinic, adrenergic, histamine, and serotonin receptors, among others [28]. In children, three main factors are primarily responsible for bronchospasm: instrumental procedures such as laryngoscopy, tracheal intubation, and laryngeal mask airway insertion, which stimulate the parasympathetic system, leading to acetylcholine release and activation of muscarinic receptors in bronchial smooth muscle, resulting in bronchospasm; histamine release triggering bronchospasm induced by certain medications, such as neuromuscular blocking agents, as well as following the administration of opioids or colloids: activation of an immune response—often anaphylactic—in reaction to agents such as latex, muscle relaxants, or other triggers, with subsequent stimulation of histamine or serotonin receptors.

Another important factor contributing to postoperative respiratory complications is residual neuromuscular block, defined as a Train-of-Four (TOF) ratio of less than 0.9. Even mild residual paralysis can impair swallowing reflexes and compromise upper airway integrity, underscoring the importance of monitoring the depth of neuromuscular blockade, including in children with URTI, although data on this population remain limited. Sugammadex, administered at a dose of 2 mg/kg in children aged 2 years and older, has demonstrated the highest efficacy in reversing neuromuscular blockade.

Intravenous lignocaine inhibits the laryngeal reflex in healthy children and may offer some benefit in children with URTI, although its effect is short-lived [29]. Topical lignocaine is not recommended for the prevention of laryngospasm in children with respiratory tract infections [30]. However, applying lignocaine gel to the laryngeal mask airway (LMA) may help reduce postoperative coughing in children with URTI [31].

### 3.3. Airways

In children with an upper respiratory tract infection (URTI) and an increased risk of adverse airway events, airway management should be performed by an experienced pediatric anesthesiologist [1]. The use of a supraglottic airway device is associated with a reduced risk of perioperative respiratory adverse events compared to the use of a tracheal tube. A face mask carries the lowest risk of adverse airway reflexes in children with URTI [3]. Whenever feasible, a face mask or laryngeal mask airway (LMA) should be used instead of endotracheal intubation [3,16]. Although the use of an LMA in pediatric patients with current or recent URTI does not completely eliminate adverse airway reflexes, it does reduce their incidence.

A randomized controlled trial by Drake-Brockman et al. demonstrated a 5.3-fold increase in the risk of perioperative respiratory adverse events in infants intubated with a tracheal tube compared with those managed with a supraglottic airway device [32]. When endotracheal intubation is required, the use of an uncuffed endotracheal tube is recommended whenever possible [3].

### 3.4. Recovery

The risk of adverse events in children with respiratory infections extends to the emergence period, particularly during the removal of airway devices [3]. Removing the LMA or endotracheal tube while the patient is still asleep may reduce the incidence of laryngospasm and other adverse respiratory events but is typically associated with an increased risk of airway obstruction [3].

The latest meta-analysis demonstrated the prophylactic potential of lidocaine, dexmedetomidine, β2-adrenoceptor agonists, and propofol induction in reducing perioperative respiratory adverse events (PRAE). However, the authors cautioned that these findings should be interpreted carefully due to inconsistent definitions of PRAE and the correlation among its individual subtypes within the composite outcome [33].

Anesthesia in children with a current respiratory infection, or one that has occurred within the previous two weeks, requires an experienced anesthesiologist due to the high risk of respiratory complications in this population. This risk decreases significantly when the interval between the resolution of infection symptoms and the planned anesthesia or surgical procedure is extended to 2–4 weeks [3]. The risk of adverse events such as bronchospasm, laryngospasm, or apnea nevertheless remains high. Walid Habre did not provide a specific recommendation regarding anesthesia in children with an active respiratory infection scheduled for elective surgery. In contrast, Britta von Ungern-Sternberg’s team supported a more permissive approach, although even mild or moderate infections necessitate anesthesia management by an experienced team. Their recommendations include the preoperative administration of salbutamol, substitution of benzodiazepines with alpha-2 agonists, intravenous induction with propofol to reduce airway reflexes, avoidance of desflurane, and the use of sevoflurane or lignocaine (excluding direct application to the vocal cords) to treat bronchospasm [34].

## 4. Conclusions

Upper respiratory tract infections are common in pediatric patients and are associated with a two- to threefold increase in the risk of perioperative respiratory adverse events. It is important to remember that in children requiring unplanned procedures—such as emergency, urgent, or immediate surgery under general anesthesia—specific guidelines should be followed to maximize safety and minimize the risk of complications. Nevertheless, most children can be safely anesthetized even when presenting with URTI symptoms, provided that preoperative, intraoperative, and postoperative management is appropriately optimized.

## Figures and Tables

**Table 1:** COLDS score.

Category	1 Point	2 Points	5 Points
Curent signs/symptoms	none	mild	moderate/severe
Onset	>4 weeks ago	2–4 weeks ago	<2 Weeks ago
Lung disease	none	mild	moderate/severe
Airway Device	none or facemask	Laryngeal mask airway or supraglottic	Tracheal tube
Surgery	Other (including ear tubes)	Minor airway	Major airway

## Data Availability

No new data were created or analyzed in this study. Data sharing is not applicable to this article.
